# A spinal cord infarction that occurred after laparoscopic gastrectomy performed under general anesthesia and epidural analgesia

**DOI:** 10.1186/s40981-017-0138-x

**Published:** 2018-01-05

**Authors:** Kei Houri, Shinichi Hamasaki, Takatoshi Tsujimoto, Tomohisa Uchida, Tatsushige Iwamoto, Toru Shirai, Shinichi Nakao

**Affiliations:** Department of Anesthesiology, Kindai University Faculty of Medicine, Ohno-Higashi, Osaka Sayama, Osaka, 589-8511 Japan

**Keywords:** Epidural analgesia, Spinal cord infarction, Paraplegia, Anterior spinal artery syndrome, Magnetic resonance imaging

## Abstract

**Background:**

Spinal cord infarction (SCI) after epidural anesthesia is quite rare. Although most cases of perioperative SCI are associated with aortic, cardiac, or spinal surgery, and/or abnormal preoperative conditions, such as spinal stenosis or hypercoagulopathy, intraoperative events, such as severe hypotension or epidural puncture and catheterization, can be contributory factors.

**Case presentation:**

A 52-year-old male was underwent laparoscopic gastrectomy. Before induction of general anesthesia, an epidural catheter was placed without any problems. The patient had no pain and no complaint just after the operation, but suddenly complained of back pain and anuria, and could not move either of his lower limbs 30 h after the operation. As we thought that the incident would be caused by the migration of the epidural catheter into the subarachnoid space, we removed the catheter, but there was no recovery of the symptoms even 20 h later. The magnetic resonance imaging (MRI) scan showed no hematoma in the epidural space but an abnormal signal within the spinal cord, extending from the Th3 to Th8 levels, which was consistent with the SCI. Unfortunately, the patient’s recovery from the paraplegia and abnormal sensation was poor.

**Conclusions:**

When a patient complains of lower limb muscle weakness and/or abnormal sensations, it is important to perform an MRI examination and treatment as early as possible to avoid permanent paraplegia, especially after epidural puncture and catheterization.

## Background

Permanent neurological deficits rarely occur after epidural anesthesia (their incidence is estimated to be ≤ 0.02%) [1]. The causes of such deficits are diverse and include spinal cord compression, direct needle trauma, neurotoxicity, or spinal cord infarctions (SCI) due to vascular issues [2]. Although most cases of perioperative SCI are associated with aortic, cardiac, or spinal surgery and are caused by abnormal conditions, such as preoperative spinal stenosis, aortic aneurysms, or hypercoagulation disorders, intraoperative events, such as severe hypotension (due to a cardiac arrest or massive hemorrhaging) and epidural analgesia, can also be contributory factors [3]. We present a case of paraplegia caused by an SCI that occurred after abdominal surgery.

## Case presentation

A 52-year-old male (89 kg, body mass index 30.1 kg/m^2^) with a stomach tumor was scheduled for laparoscopic gastrectomy. His medical history included hypertension, diabetes mellitus, hyperlipidemia, and smoking.

In the operating room, after the placement of standard monitors, a Tuohy needle was inserted into the epidural space at the Th8–9 level while the patient was in the right lateral position. The needle was guided into position based on the orientation of the bevel of its cephalad. An epidural catheter was inserted 5 cm into the epidural space. No blood or cerebrospinal fluid (CSF) was aspirated from the catheter. Then, 3 ml of 1% mepivacaine was injected through the catheter without a motor block. General anesthesia was induced with 110 mg propofol and remifentanil, and the trachea was intubated with the aid of 50 mg rocuronium. Anesthesia was maintained with an oxygen/air mixture and 1.5–2% sevoflurane. Six milliliters of 1% mepivacaine was injected into the epidural space 10 min before the operation, and a continuous infusion of 140 ml of 0.25% levobupivacaine, 0.5 mg fentanyl, and 50 ml saline into the epidural space was started at a rate of 4 ml/h. The patient’s vital signs remained stable throughout the operation and he did not suffer severe hypotension. Blood loss was minimal. At the completion of the operation, the patient was extubated, and we confirmed that there were no signs of lower limb muscle weakness.

**Fig. 1:**
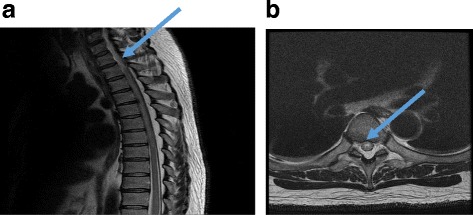
**a** Sagittal T2-weighted magnetic resonance imaging (MRI) scan through the thoracic spinal cord obtained on postoperative day 2 showing abnormal T2 high signal intensity from the Th3 to Th8 levels (arrow). **b** Axial T2-weighted image showing high signal intensity in the anterior horn of the gray matter (owl’s eye) at the Th5 level (arrow)

Good analgesia was achieved, and the patient did not suffer any postoperative complications associated with the epidural anesthesia. On postoperative day (POD) 1 (approximately 24 h after the operation), as the patient was able to walk and urinate without difficulty, the urinary catheter was removed. However, at 30 h after the operation, he suddenly developed back pain and anuria and could not move either of his lower limbs. The patient’s touch sensations became dull below his nipples. Although no CSF was aspirated from the catheter, we assumed that the epidural catheter had moved into the subarachnoid space and stopped the epidural infusion. Two hours later, the patient was able to move both of his ankles and felt that his touch sensation was improved. However, on POD 2, his knee movement was still inhibited, and therefore, we removed the epidural catheter and performed a magnetic resonance imaging (MRI) scan of his spinal cord. The T2-weighted images of MRI scan showed an abnormal signal within the spinal cord, extending from the Th3 to Th8 levels, which was consistent with an SCI (Fig. 1a, b), but no hematoma or abscess (which could have compressed the spinal cord) was found. Especially, there was the “owl’s eye” appearance in the axial view (Fig. 1b), which is specific for infarction of the anterior horn [2]. Diffusion weighted imaging (DWI) also showed hyperintensity areas, which corresponded with T2-weighted imaging, and apparent diffusion coefficient (ADC) was decreased (data not shown). We planned to perform angiography in order to investigate the exact cause of the infarction, but the patient did not consent to it. Based on the patient’s MRI findings and advice from orthopedists and neurosurgeons, 300 mg hydrocortisone and Edaravone®, an antioxidant, were administered intravenously, but no anticoagulant therapy was conducted due to a fear of postoperative bleeding. Unfortunately, the patient’s paraplegia and sensation problems did not improved much. On POD 23, the patient was transferred to another hospital to undergo rehabilitation.

### Discussion

The etiologies of post-epidural anesthesia SCI include direct compression of the spinal cord by a hematoma or abscess, and ischemia of the spinal cord secondary to hypotension, spasm, an embolism, arterial dissection, or trauma involving the important arteries that feed the spinal cord [4]. In the present case, no spinal cord compression by a hematoma was found, and there was no abnormal perioperative hypotension. Taken together, the most plausible cause of the SCI seen in the current case seems to be occlusion of the important arteries that feed the spinal cord, specifically the anterior spinal artery (ASA).

The spinal cord is perfused by a single anterior and two posterior spinal arteries. The ASA arises from the vertebral artery, descends the length of the spinal cord, and supplies blood to the anterior two thirds of the spinal cord. However, as the vertical connectivity of the ASA is poor, the areas of the spinal cord supplied by the ASA are vulnerable to ischemia. Occlusion of the ASA produces a clinical condition known as ASA syndrome. Its clinical features include motor paralysis, areflexia, and the loss of bowel and bladder sphincter function, and sensory changes include the absence of pain and temperature sensation, while proprioception and the ability to perceive vibrations are unimpaired due to the sparing of the posterior columns. The symptoms and signs seen in our case agree well with all of these clinical features of ASA syndrome. MRI is a reliable and sensitive means of evaluating SCI, which produce abnormal T2 hyperintensity and enhancement on such scans [2]. Tumors, myelitis, and demyelination also produce abnormal T2 hyperintensity, but in the present case, we were able to rule out those because of the clinical course of the patient’s conditions and “owl’s eye” appearance in the axial view of T2-weighted imaging (Fig. 1b). Diffusion weighted imaging (DWI) is recognized as a sensitive and useful tool to detect acute SCI [5]. In DWI, SCI can be detected as early as 3 h after the onset, while it takes around 24 h for T2-weighted imaging to diagnose SCI [5]. We should have performed MRI, especially DWI, when the patient first complained of lower limb muscle weakness and a urinary disorder on POD 1. Hong et al. reported a case in which ASA syndrome occurred following lumbar epidural anesthesia for total hip arthroplasty and speculated that the cause of the ASA syndrome was a combination of intraoperative hypotension, the patient’s position, osteophytic narrowing of the intervertebral foramina, and adrenaline-induced vasoconstriction [4]. Hobai et al. [3] proposed the following risk factors for perioperative SCI: preexisting medical conditions (spinal stenosis, an aortic aneurysm, vasculitis, advanced age, pregnancy, and malignancy), epidural anesthesia (e.g., the administration of adrenaline and the rapid infusion of a large amount of solution), and intraoperative events (e.g., hypotension, extreme positioning, and vascular compression). In the present patient, intraoperative hypotension, spinal cord compression by an epidural hematoma, etc. were excluded as potential etiologies of the SCI. In addition, we did not administer adrenalin or a large amount of solution into the epidural space. Judging from the later onset of paraplegia and the lack of hematoma in the epidural space, occlusion of the feeding arteries of the ASA by a thromboembolism seems to be the most plausible explanation for the patient’s SCI. Indeed, the patient suffered from hypertension, diabetes mellitus, malignancy, and hyperlipidemia, all of which are risk factors for thromboembolisms. However, Akaishi et al. [6] have recently proposed that epidural needles and catheters can directly damage the spinal cord-nourishing arteries (including the anterior radicular arteries). According to their hypothesis, the persistent compression of a spinal cord-nourishing artery by an epidural catheter cannot be completely ruled out in the current case. Unfortunately, we were not able to examine this hypothesis because the epidural catheter was pulled out when we acquired the MRI scan, and angiography of the spinal cord was not performed. Although the infarction level (Th3 to Th8) was a little higher than the level at which the epidural puncture was actually performed (Th8–9), the catheter could reach those areas because it was inserted and advanced based on the position of the needle’s cephalad.

Anyhow, it was impossible to identify a single cause of the ASA syndrome seen in the present case. However, when a patient complains of lower limb muscle weakness and/or abnormal sensation, it is important to perform an MRI examination and treatments as soon as possible to avoid permanent paraplegia, especially following epidural analgesia.

## Conclusions

We describe our experience with a patient who had persistent paraplegia and sensation problems due to a spinal cord infarction after laparoscopic gastrectomy performed under general anesthesia and epidural analgesia. When a patient complains of abnormal muscle weakness and/or sensation following epidural analgesia, it is important to perform an MRI examination as soon as possible to avoid permanent paraplegia.

## Abbreviations

ASA: Anterior spinal artery
CSF: Cerebrospinal fluid
MRI: Magnetic resonance imaging
POD: Postoperative day
SCI: Spinal cord infarction

## References

[1] Kane RE (1981). Neurologic deficits following epidural or spinal anesthesia. Anesth Analg.

[2] Chan LL, Kumar AJ, Leeds NE, Forman AD (2002). Post-epidural analgesia spinal cord infarction. Acta Neurol Scand.

[3] Hobai IA, Bittner EA, Grecu L (2008). Perioperative spinal cord infarction in nonaortic surgery. J Clin Anesth.

[4] Hong HD, Lawrence HM (2001). Anterior spinal artery syndrome following total hip arthroplasty under epidural anesthesia. Anesth Intensive Care.

[5] Tsang BK, Foster E, Kam A, Storey E (2013). Diffusion weighted imaging with trace diffusion weighted imaging, the apparent diffusion coefficient and exponential images in the diagnosis of spinal cord infarction. J Clin Neurosci.

[6] Akaishi S, Koakutsu T, Yamauchi M (2015). Pathomechanisms and treatment of paraplegia after epidural anesthesia. Anesth Resusc.

